# Fauna of nocturnal moth species collected in a semi-natural grassland at Kanpu-zan in northern Japan

**DOI:** 10.3897/BDJ.7.e37968

**Published:** 2019-08-02

**Authors:** Masaru Kamikura, Yuzu Sakata

**Affiliations:** 1 Department of Biological Environment, Faculty of Bioresource Sciences, Akita Prefectural University, Akita, Japan Department of Biological Environment, Faculty of Bioresource Sciences, Akita Prefectural University Akita Japan; 2 Sugadaira Research Station, Mountain Science Center, University of Tsukuba, Nagano, Japan Sugadaira Research Station, Mountain Science Center, University of Tsukuba Nagano Japan

**Keywords:** arthropods, northern Japan, nocturnal moth, semi-natural grassland, species occurences

## Abstract

Semi-natural grasslands, which house species-rich ecosystems, have rapidly declined since the twentieth century due to land-use practices, such as agricultural intensification and abandonment. Owing to their diversity and known habitat associations, nocturnal moths are considered as one of the most suitable organisms to be studied for assessing the dynamics of species composition as a result of changes in landscape management of semi-natural grasslands. The present study provides the foremost description of nocturnal moth fauna of the semi-natural grassland at Kanpu-zan, northern Japan. Moth population data from 1987 were compared to the data collected in 2018 to evaluate the impact of decline in grasslands on species-richness. During the field sampling in 2018, a total of 226 nocturnal moth species were detected, which was nearly two-thirds of the number of species recorded in 1987, i.e. 396 species. The values obtained in 2018 were found to be nearly constant for different sites. For both periods, it was evident that moth fauna in Kanpu-zan mainly consisted of species that relied on woody plants. Amongst the species which were only recorded in 1987, 107 species were generalists that fed on plants that are commonly distributed in Kanpu-zan. No moth species were recorded that depended upon endangered or extinct plant food sources. Thus, it is unlikely that the decline in the number of moth species in Kanpu-zan was due to the loss in plant food sources. Our results suggest that environmental factors other than food plants may have caused decline and changes in nocturnal moth fauna. More studies on various organism fauna are needed for understanding the conservation of semi-natural grassland, considering that the loss of semi-natural grasslands is one of the major threats to biodiversity.

## Introduction

Semi-natural grasslands house species-rich ecosystems including regional meta-communities by specialists of open habitats (Emanuelsson 2008, Pykälä et al. 2005, Alison et al. 2017). The persistence of semi-natural grassland depends on anthropogenic activities, such as mowing, grazing and burning (Suka et al. 2012, Dengler et al. 2014, Ushimaru et al. 2018). Recent studies have shown that the grasslands may have persisted during the Holocene in a natural state due to natural disturbances and severe environmental conditions (Nakahama et al. 2018, Ohwaki 2018). However, these semi-natural grasslands have shown a continuous and rapid decline since the twentieth century due to changes in land-use practices such as agricultural intensification and abandonment (Donald et al. 2001, Eriksson et al. 2002, Koyanagi and Furukawa 2013). As a result, many grassland species, including insects feeding on grassland plants and those inhabiting grassland habitat, are presently endangered due to the scarcity of natural grasslands in these regions (Maes and Van Dyck 2001, Habel et al. 2013). There is an urgent need to understand the changes in grassland fauna due to human activities.

Nocturnal moths are an ecologically important group of insects that play a key role in herbivory and pollination. They act as a food source for birds as well as potential indicators of ecosystem change across a wide variety of landscapes (Kitching et al. 2000, Summerville and Crist 2004, Macgregor et al. 2016). Declines in moth populations have been linked to habitat loss and agricultural intensification (Wenzel et al. 2006, Merckx et al. 2012). Owing to their diversity and known habitat associations, nocturnal moths are considered as one of the most suitable study taxa for assessing species richness against the changes in landscape management of semi-natural grasslands (Erhardt and Thomas 1991, Wenzel et al. 2006; Alison et al. 2017).

Kanpu-zan, which is located in northern Japan, consists of semi-natural grasslands partly maintained by mowing and burning and accommodates many endangered grassland species (Akita Prefecture 2016). The region was a rich source of grassland resources as a livestock feed for farmers until the 1960s. However, the utilisation of the grassland, such as the grass harvest, gradually declined leading to succession by dense shrubs and trees from the 1970s as lifestyles changed. A study indicates that the grassland area, which was 319 ha in 1975, had decreased to 138 ha by 2014 (Masui et al. 2017). The prevalent plant community varied depending on the management frequency and plant richness was lower in abandoned sites with reduced or diminished grassland species (Masui et al. 2017). Documentation of moth species in the region was conducted in 1987 before the grassland had severely declined (Takahashi 1993). However, the present status of moth fauna is largely unknown. This study presents the first account of nocturnal moths in Kanpu-zan after the decline in grasslands and presents a model for understanding the changes in moth fauna related to altered vegetation in grassland areas caused by changes in human activity. Uchida and Ushimaru (2014) compared plant richness and butterfly and orthopteran richness and diversity amongst three different land use types in semi-natural grasslands: abandoned, traditional and intensified terraces and suggested that the number of individuals of most herbivorous species decreased randomly with respect to life-history traits, following a decline in plant richness reflecting disturbance frequency. Due to the present plant flora being revealed in Kanpu-zan, this site provides a promising opportunity to understand the relationship between moth fauna and the land use of the grassland vegetation.

## Materials and Methods

### Study site

**Figure 1.**
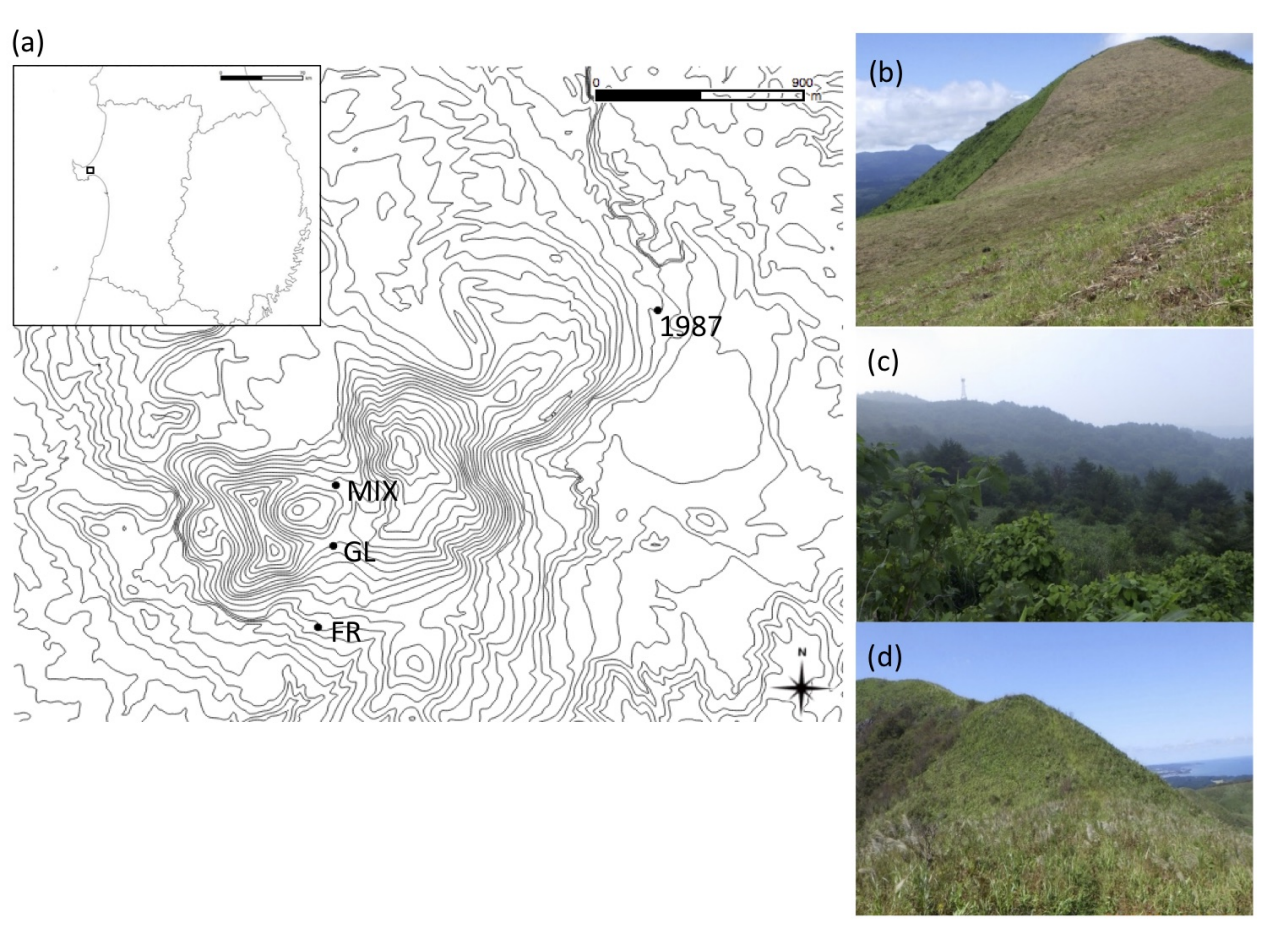
Studied area and habitat vegetation: **a.** Geographic location of Kanpu-zan; **b.** grassland (GL); **c.** forest (FR); **d.** mixed vegetation (MIX).

Surveys of nocturnal moths were carried out at Kanpu-zan during May to October in 2018, twice a month (12 nights in total), on clear nights from sunset to midnight. Three sites that differ in vegetation were selected: 1) grassland maintained by mowing once or twice a year (grassland site), 2) site where grassland management had been abandoned leading to overgrowth of shrubs and trees (forest site) and 3) mixed vegetation with both grassland and woody plants (mixed site) (Fig. 1). In 1987, the moths were collected during May to September, once or twice a month (6 nights in total) at one site in a grassland dominated by tall grass such as *Miscanthus sinensis*, where shrubs and hedgerow trees were interspersed (Takahashi 1993).

### Sampling methods

Moths were collected using UV light traps, equipped with a 20 W black (ultraviolet) light fluorescent tube. One light trap was set at each site. The same method was used in 1987 (Takahashi 1993). All collected specimens were dried at room temperature and mounted for morphological examination. All moths were identified to species level by M. Kamikura and K. Umetsu using descriptions and photos in the book "Standard of Moths in Japan" (Hirowatari et al. 2013, Kishida 2011a, Kishida 2011b, Nasu et al. 2013). Some of the family names (i.e. Arctiidae, Lymantriidae, Micronoctuidae) were updated to current taxonomy. All the specimens were preserved as a personal collection of M. Kamikura.

### Data analyses

Owing to the annual variations in abundance of moth species (Highland et al. 2013), data analyses were based on presence-absence data of moths sampled at each site in each month (Table 1,Suppl. material 1). Species composition was compared between years and amongst sites. In addition, because micro-moths belonging to the families of Chrysopeleiidae, Cosmopterigidae, Oecophoridae and Depressariidae were not identified in the study in 1987 (Takahashi 1993), those specimens were excluded from the data in 2018, as well as from the following analysis. In order to test whether the community composition of moth species differed between 1987 and 2018, permutational multivariate analyses of variance (PERMANOVA) were performed. We included the year of the survey as an explanatory variable. Additionally, PERMANOVA was also performed to test whether the community composition of moth species differed amongst the three sites in 2018. Here each site was included as an explanatory variable. For these analyses, the ADONIS function in library VEGAN (Oksanen et al. 2016) in R. ver. 3.3.2 (R Development Core Team 2016) was used. Non-metric multidimensional scaling analysis (NMDS) on the Jaccard index was used to visually summarise the plant community compositions of each site at each month.

In order to explore the food habits of moth species at each site in both years, the moth species were classified into six feeding groups: 1) species that primarily feed on herbs, 2) species that primarily feed on shrubs and/or trees, 3) species that feed on both herbs and trees, 4) species that primarily feed on lichens, 5) species that primarily feed on dead leaves and 6) species whose food habit is unknown, referring to the guide “The Standard of Moths in Japan I-IV” (Kishida 2011a, Kishida 2011b, Hirowatari et al. 2013, Nasu et al. 2013). The collected moths were also classified into two groups based on the range of host plants: 1) specialist, defined as species that feed on only one plant family and 2) generalists, defined as species that feed on more than two plant families. We calculated the proportions of each feeding groups for each site in both years.

## Results and Discussion

**Figure 2.**
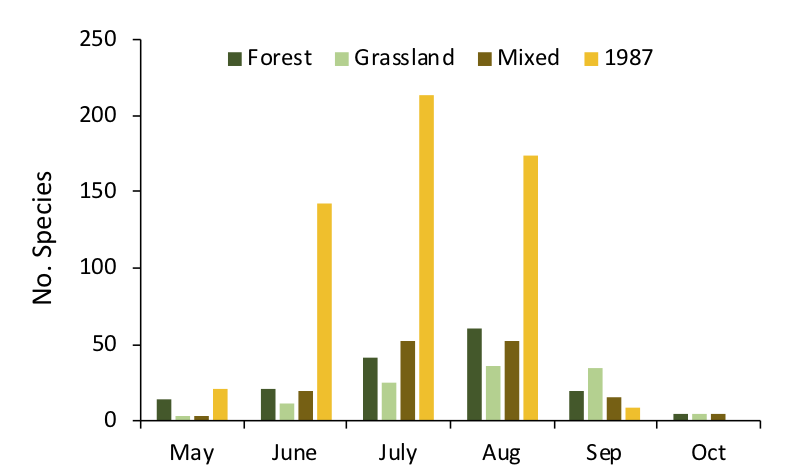
Number of moths collected in each site in both years in each month.

The number of taxa of moths recorded in 2018 at each site (excluding the 4 families and 10 species of micro-moths) was 14 families, 79 genera and 95 species for the grassland site; 16 families, 115 genera and 137 species for the forest site and 14 families, 103 genera and 123 species for the mixed site (Fig. 2). In total, 17 families, 181 genera and 226 species were recorded in 2018. This was nearly two-thirds of the species recorded in 1987, which comprised of 16 families, 291 genera and 396 species. Amongst all the moth species documented, only 118 species were observed in both periods. The 278 species that were recorded in 1987 were not recorded in 2018, while 115 species were newly recorded in 2018.

### Species composition of moths between years and amongst sites

**Figure 3.**
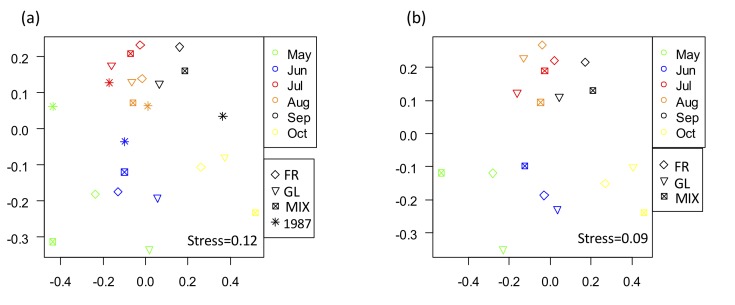
Non-metric multidimensional scaling (NMDS) ordination graphs of monthly aggregated moth communities showing (a) for both years and (b) for 2018.

The results of the PERMANOVA showed that the species composition of the moths significantly differed between years (Fig. 3a; R^2^ = 0.06, P = 0.02). This suggests that species composition at the three sites in 2018 differed from that in 1987. On the other hand, when the data of 1987 was excluded from the analysis, it did not differ amongst sites in 2018 (Fig. 3b; R^2^ = 0.08, P = 0.99). This may be because the three sites in 2018 were located in a geographically adjacent area (an average of 400 m between sites). Studies that detected differences in moth species composition amongst different vegetation and management were sites that were geographically separated by more than 1 km (Öckinger and Smith 2006, Pöyry et al. 2009). As some moth species are known to show high mobility (i.e. a flight range larger than 500 m in radius) (van der Meulen and Groenendijk 2005, Merckx and Macdonald 2015), species composition of moths may not differ in a small spatial scale. Furthermore, because the present data included only one replicate for each type of vegetation, more replicates from multiple sites would be useful in confirming that the difference in vegetation reflecting the management did not affect the moth species composition in the semi-natural grassland in Kanpu-zan.

### Food habits

**Figure 4.**
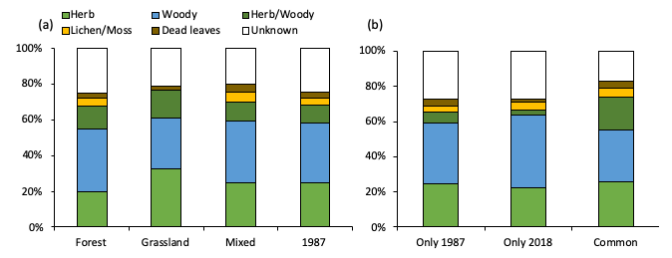
Proportion of each food habits for moth species (a) of each site in both years, (b) those unique to each year and common in both years.

The number of species that may rely on woody plants comprised more than half of the total number of species at all sites in both years (Fig. 4a). This includes species that feed only on woody plants; species that feed on both herbs and woody plants; species that feed on lichens and mosses; and species that feed on dead leaves. These species were known to consume woody plants that belong to the families of Fagaceae, Rosaceae, Fabaceae and Caprifoliaceae. Trees and shrubs that belong to Fagaceae and Rosaceae were dominant in the forest site and Fabaceae shrubs and vines were scattered across the grassland site, while the mixed site had a mixture of vegetation of both sites. Moreover, trees and shrubs such as *Cerasus jamasakura* H.Ohba and *Quercus serrata* Murray were present in grasslands in 1987 (Takahashi 1993, K. Umetsu, personal observation). These suggest that, in both years, moth fauna in Kanpu-zan was mainly comprised of species that may feed on woody plants.

For the 118 species that were recorded in both in 1987 and 2018, the composition rate of species that may use both herbs and woody plants was 19%, which was higher compared to that for the species that were only recorded in either 1987 or 2018, at 10.1% and 11.2%, respectively (Fig. 4b). This may be because the species that can feed on both herbs and woody species are adapted to habitats with various vegetations and were tolerant to the environmental change between 1987 and 2018 caused by decline in grasslands. Species that primarily feed on herbs such as *Deilephila askoldensis*, which is known to inhabit volcanic grasslands (Yano 2011), consisted of 15-20% in all three sites in 2018. The semi-natural grasslands in Kanpu-zan may be a valuable habitat for the larvae of these moths. On the other hand, the composition rate of species that feed on lichens was relatively low in all sites and especially low at the grassland site in 2018 (Fig. 4a). This is consistent with the result in 1987, which identified that it is one of the characteristics in grasslands that lichen feeding moths are scarce (Takahashi 1993).

**Figure 5.**
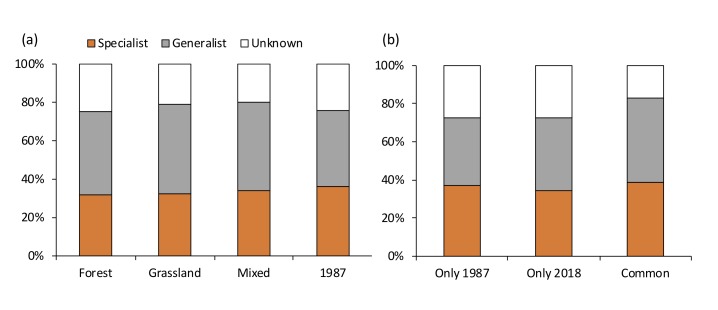
Proportion of food range (specialist/generalist) for moth species (a) of each site in both years, (b) those unique to each year and common in both years.

The composition rate of specialist and generalist moths was stable across sites including the year 1987. Generalists consisted of 43.8±1.5% (mean±SE) of the total number of moth species in both years (Fig. 5). Out of the 280 species of moths that were only recorded in 1987, 107 species were generalists that feed on plants that are commonly distributed in Kanpu-zan (Masui et al. 2017).

## Conclusion

The present study suggests the species composition changed between 1987 and 2018 and the number of moth species largely declined in the last 30 years. Wenzel et al. (2006) also showed that the number of butterflies and burnet moths had declined between 1972 and 2001 in calcareous grassland remnants in south-western Germany. They suggested that species which require structured habitats, species with low mobility, species which require more than 16 ha of habitat and specialist feeders were especially affected by the decline. As we did not find any moths that depend on endangered plants or whose food plant had become extinct, it is unlikely that the decline in the moths in Kanpu-zan was due to the loss in food plants. Other factors such as plant height, flowering species richness and habitat connectivity (Pöyry et al. 2009) may have caused the decline. Continuing landscape management such as mowing and burning may be important, not only for endangered species, but also for common moth species and to maintain the nocturnal moth community in semi-natural grasslands.

A previous study in Japan covering 31 agricultural areas, including semi-natural grasslands, demonstrated that declines in herbivorous insects in both abandoned and intensified use of agricultural landscapes are also explained by multiple factors (Uchida and Ushimaru 2014). It has been reported that there are large differences with respect to the preferred management intensity amongst species groups in the grassland habitats (Pöyry et al. 2006). More studies on other organism fauna are needed for understanding the conservation of semi-natural grassland, considering that the loss of semi-natural grasslands is one of the major threats to biodiversity.

## Supplementary Material

621e0ca5-0953-5585-8085-fb1dbc67b279 10.3897/BDJ.7.e37968.suppl1 Supplementary material 1 monthly species occurences Data type: occurrences
Brief description: monthly species occurrences of nocturnal moths
File: oo_320380.csv https://binary.pensoft.net/file/320380 Masaru Kamikura, Yuzu Sakata

## Figures and Tables

**Table 1.** Species list (presence (1) or absence (0)) of the three sites in 2018 and one site in 1987.

**family**	**species name**	**food**	**specialist/generalist**	**1987**	**forest**	**grassland**	**mixed**
** Bombycidae **	*Bombyx mandarina* (Moore, 1872)	woody	specialist	0	1	1	0
** Chrysopeleiidae **	*Ascalenia* sp. (Japanese name: Zugurokobukazariba)	unknown	unknown	0	0	0	1
** Cosmopterigidae **	*Pyroderces sarcogypsa* (Meyrick, 1932)	unknown	unknown	0	0	0	1
** Cosmopterigidae **	*Ressia quercidentella* Sinev, 1988	unknown	unknown	0	1	0	1
** Cossidae **	*Pharagmataecia castaneae* (Hübner, 1790)	herb	specialist	1	0	0	0
** Cossidae **	*Zeuzera multistrigata* Moore, 1881	woody	generalist	0	1	1	1
** Crambidae **	*Agrotera posticalis* Wileman, 1911	woody	specialist	0	0	0	1
** Crambidae **	*Anania albeoverbascalis* Yamanaka, 1966	unknown	unknown	1	0	0	0
** Crambidae **	*Anania verbascalis* ([Denis & Schiffermüller], 1775)	herb	specialist	1	0	0	0
** Crambidae **	*Ancylolomia japonica* Zeller, 1877	herb	specialist	0	0	0	1
** Crambidae **	*Bradina angustalis* Yamanaka, 1984	unknown	unknown	1	0	0	0
** Crambidae **	*Bradina atopalis* (Walker, 1859)	unknown	unknown	0	1	1	1
** Crambidae **	*Calamotropha okanoi* Bleszynski, 1961	unknown	unknown	1	0	0	0
** Crambidae **	*Camptomastix hisbonalis* (Walker, 1859)	unknown	unknown	0	1	0	0
** Crambidae **	*Chilo luteellus* (Motschulsky, 1866)	herb	generalist	0	0	0	1
** Crambidae **	*Chilo suppressalis* (Walker, 1863)	herb	specialist	1	0	0	0
** Crambidae **	*Chrysoteuchia diplogramma* (Zeller, 1863)	herb	specialist	0	1	0	0
** Crambidae **	*Circobotys nycterina* Butler, 1879	unknown	unknown	1	0	0	0
** Crambidae **	*Cnaphalocrocis medinalis* (Guenée, 1854)	herb	specialist	1	0	0	0
** Crambidae **	*Cnaphalocrocis stereogona* (Meyrick, 1886)	herb	specialist	1	1	0	0
** Crambidae **	*Crambus argyrophorus* Butler, 1878	unknown	unknown	1	0	0	0
** Crambidae **	*Crambus perlellus* (Scopoli, 1763)	herb	specialist	1	0	0	0
** Crambidae **	*Daulia afralis* Walker, 1859	herb	specialist	1	0	0	0
** Crambidae **	*Diasemia reticularis* (Linnaeus, 1761)	herb	generalist	1	0	0	0
** Crambidae **	*Dolicharthria bruguieralis* (Duponchel, 1833)	herb	generalist	1	0	0	0
** Crambidae **	*Flavocrambus striatellus* (Leech, 1889)	unknown	unknown	1	0	0	0
** Crambidae **	*Glaucocharis exsectella* (Christoph, 1881)	lichen/moss	specialist	1	0	0	1
** Crambidae **	*Glyphodes pryeri* Butler, 1879	woody	specialist	1	0	0	0
** Crambidae **	*Haritalodes derogatus* (Fabricius, 1775)	woody	specialist	0	0	0	1
** Crambidae **	*Herpetogramma luctuosale* (Guenée, 1854)	woody	specialist	1	1	1	1
** Crambidae **	*Herpetogramma magnum* (Butler, 1879)	herb/woody	generalist	0	0	0	1
** Crambidae **	*Herpetogramma moderatale* (Christoph, 1881)	herb	generalist	1	0	0	0
** Crambidae **	*Herpetogramma stultale* (Walker, 1859)	herb	generalist	1	0	0	0
** Crambidae **	*Mabra charonialis* (Walker, 1859)	dead leaves	generalist	1	0	0	0
** Crambidae **	*Maruca vitrata* (Fabricius, 1787)	herb	specialist	1	0	0	0
** Crambidae **	*Microchilo inouei* Okano, 1962	unknown	unknown	1	0	0	0
** Crambidae **	*Nacoleia commixta* (Butler, 1879)	woody	generalist	1	1	0	0
** Crambidae **	*Nacoleia sibirialis* (Millière, 1879)	unknown	unknown	1	0	0	0
** Crambidae **	*Nacoleia tampiusalis* (Walker, 1859)	unknown	unknown	0	0	1	1
** Crambidae **	*Nomis albopedalis* Motschulsky, 1861	unknown	unknown	1	1	1	1
** Crambidae **	*Nomophila noctuella* (Denis & Schiffermüller, 1775)	herb/woody	generalist	1	0	0	0
** Crambidae **	*Omiodes tristrialis* (Bremer, 1864)	unknown	unknown	1	0	0	1
** Crambidae **	*Ostrinia latipennis* (Warren, 1892)	herb	specialist	1	0	0	0
** Crambidae **	*Palpita nigropunctalis* (Bremer, 1864)	woody	generalist	0	1	1	1
** Crambidae **	*Patissa fulvosparsa* (Butler, 1881)	unknown	unknown	0	0	0	1
** Crambidae **	*Piletocera sodalis* (Leech, 1889)	unknown	unknown	1	0	0	0
** Crambidae **	*Platytes ornatella* (Leech, 1889)	unknown	unknown	1	0	0	0
** Crambidae **	*Pleuroptya chlorophanta* (Butler, 1878)	woody	generalist	1	0	0	0
** Crambidae **	*Pleuroptya harutai* (Inoue, 1955)	woody	specialist	0	0	1	0
** Crambidae **	*Pleuroptya inferior* (Hampson, 1898)	herb	specialist	1	0	0	0
** Crambidae **	*Prodasycnemis inornata* (Butler, 1879)	herb	specialist	1	0	0	0
** Crambidae **	*Pyrausta unipunctata* Butler, 1881	unknown	unknown	1	0	0	0
** Crambidae **	*Scirpophaga virginia* Schultze, 1908	unknown	unknown	1	0	0	0
** Crambidae **	*Sitochroa palealis* (Denis & Schiffermüller, 1775)	herb	specialist	0	0	1	0
** Crambidae **	*Sitochroa umbrosalis* (Warren, 1892)	herb	specialist	1	1	1	1
** Crambidae **	*Sitochroa verticalis* (Linnaeus, 1758)	herb	generalist	0	0	1	1
** Crambidae **	*Spoladea recurvalis* (Fabricius, 1775)	herb	generalist	1	1	1	0
** Crambidae **	*Syllepte pallidinotalis* (Hampson, 1912)	woody	generalist	1	0	0	0
** Crambidae **	*Syllepte segnalis* (Leech, 1889)	unknown	unknown	1	0	0	0
** Crambidae **	*Torulisquama obliquilinealis* Inoue, 1982	unknown	unknown	1	0	0	0
** Crambidae **	*Tyspanodes striatus* (Butler, 1879)	woody	generalist	1	0	0	0
** Crambidae **	*Udea lugubralis* (Leech, 1889)	unknown	unknown	1	0	0	0
** Crambidae **	*Uresiphita gracilis* (Butler, 1879)	unknown	unknown	1	0	0	0
** Crambidae **	*Xanthocrambus lucellus* (Herrich-Schäffer, 1848)	unknown	unknown	0	1	1	0
** Depressariidae **	*Agonopterix mutuurai* Saito, 1980	herb	specialist	0	0	0	1
** Depressariidae **	*Agonopterix sumizome* Fujisawa, 1985	herb	specialist	0	0	0	1
** Depressariidae **	*Agonopterix yomogiella* Saito, 1980	herb	specialist	0	0	0	1
** Drepanidae **	*Habrosyne pyritoides* (Butler, 1766)	woody	specialist	1	0	0	0
** Drepanidae **	*Tethea ampliata* (Butler, 1878)	woody	specialist	1	0	0	0
** Drepanidae **	*Thyatira batis* (Linnaeus, 1758)	woody	specialist	1	0	0	0
** Erebidae **	*Arctornis l-nigrum* (Müller, 1764)	woody	generalist	0	1	0	0
** Erebidae **	*Artaxa subflava* (Bremer, 1864)	herb/woody	generalist	1	1	1	1
** Erebidae **	*Barsine aberrans* (Butler, 1877)	lichen/moss	generalist	0	1	0	0
** Erebidae **	*Barsine pulchra* (Butler, 1877)	unknown	unknown	1	0	1	0
** Erebidae **	*Barsine striata* (Bremer & Grey, 1853)	dead leaves	generalist	1	0	0	1
** Erebidae **	*Chinoarctia nivea* (Ménétriés, 1859)	herb	generalist	1	1	1	1
** Erebidae **	*Cifuna locuples* Walker, 1855	herb/woody	generalist	1	0	0	0
** Erebidae **	*Cyana hamata* (Walker, 1854)	lichen/moss	generalist	1	0	0	1
** Erebidae **	*Eilema affineola* (Bremer, 1864)	unknown	unknown	1	1	1	1
** Erebidae **	*Eilema deplena* (Esper, 1787)	lichen/moss	generalist	1	1	0	0
** Erebidae **	*Eilema japonica* (Leech, 1889)	lichen/moss	generalist	0	1	0	0
** Erebidae **	*Eilema nankingica* (Daniel, 1954)	unknown	unknown	1	0	0	0
** Erebidae **	*Eilema vetusta* (Walker, 1854)	lichen/moss	generalist	1	1	0	1
** Erebidae **	*Epatolmis caesarea* (Goeze, 1781)	herb/woody	generalist	1	1	1	0
** Erebidae **	*Ghoria collitoides* Butler, 1885	unknown	unknown	1	1	1	1
** Erebidae **	*Ghoria gigantea* (Oberthür, 1879)	woody	generalist	0	1	0	1
** Erebidae **	*Ilema eurydice* (Butler, 1885)	woody	generalist	1	0	1	0
** Erebidae **	*Kidokuga piperita* (Oberthür, 1880)	woody	generalist	1	0	0	1
** Erebidae **	*Lemyra imparilis* (Butler, 1877)	woody	generalist	1	0	0	0
** Erebidae **	*Lemyra inaequalis* (Butler, 1879)	woody	generalist	1	0	0	0
** Erebidae **	*Lithosia quadra* (Linnaeus, 1758)	lichen/moss	generalist	1	0	0	0
** Erebidae **	*Lymantria mathura* (Moore, 1865)	woody	generalist	1	1	0	0
** Erebidae **	*Miltochrista calamina* Butler, 1877	woody	generalist	1	1	1	1
** Erebidae **	*Miltochrista miniata* (Forster, 1771)	lichen/moss	generalist	1	1	0	1
** Erebidae **	*Mimachrostia fasciata* Sugi, 1982	unknown	unknown	1	1	0	0
** Erebidae **	*Orgyia thyellina* Butler, 1881	herb/woody	generalist	0	1	0	0
** Erebidae **	*Pelosia muscerda* (Hufnagel, 1766)	lichen/moss	generalist	1	0	0	1
** Erebidae **	*Pelosia noctis* (Butler, 1881)	lichen/moss	generalist	1	0	0	0
** Erebidae **	*Pelosia obtusa* (Herrich-Schäffer, 1852)	unknown	unknown	1	0	0	0
** Erebidae **	*Phragmatobia amurensis* Seitz, 1910	herb	generalist	1	0	0	0
** Erebidae **	*Rhyparioides amurensis* (Bremer, 1861)	herb	specialist	1	1	1	1
** Erebidae **	*Rhyparioides metelkana* (Lederer, 1861)	herb	generalist	1	0	1	0
** Erebidae **	*Rhyparioides nebulosa* Butler, 1877	herb	generalist	1	1	0	1
** Erebidae **	*Sphrageidus similis* (Fuessly, 1775)	woody	generalist	1	1	0	0
** Erebidae **	*Spilarctia lutea* (Hufnagel, 1766)	unknown	unknown	1	0	0	0
** Erebidae **	*Spilarctia obliquizonata* (Miyake, 1910)	woody	specialist	0	1	0	0
** Erebidae **	*Spilarctia seriatopunctata* (Motschulsky, 1861)	woody	generalist	1	0	1	1
** Erebidae **	*Spilosoma lubricipedum* (Linnaeus, 1758)	woody	generalist	1	0	0	0
** Erebidae **	*Spilosoma punctarium* (Stoll, 1782)	woody	generalist	1	1	1	0
** Erebidae **	*Stigmatophora rhodophila* (Walker, 1865)	lichen/moss	generalist	1	0	0	0
** Erebidae **	*Thumatha ochracea* (Bremer, 1861)	unknown	unknown	1	0	0	0
** Eupterotidae **	*Apha aequalis* (Felder, 1874)	herb/woody	generalist	1	1	1	1
** Geometridae **	*Abraxas fulvobasalis* Warren, 1894	woody	specialist	1	0	0	0
** Geometridae **	*Abraxas miranda* Butler, 1878	woody	specialist	1	0	0	0
** Geometridae **	*Abraxas niphonibia* Wehrli, 1935	woody	specialist	1	0	1	0
** Geometridae **	*Acrodontis fumosa* (Prout, 1930)	woody	specialist	0	0	0	1
** Geometridae **	*Amraica superans* (Butler, 1878)	woody	specialist	1	1	1	0
** Geometridae **	*Arichanna melanaria* (Linnaeus, 1758)	woody	specialist	1	0	0	0
** Geometridae **	*Ascotis selenaria* (Denis & Schiffermüller, 1775)	herb/woody	generalist	1	0	0	0
** Geometridae **	*Astygisa chlororphnodes* (Wehrli, 1936)	woody	specialist	1	1	0	0
** Geometridae **	*Chariaspilates formosaria* (Eversmann, 1837)	herb/woody	generalist	0	1	1	1
** Geometridae **	*Chiasmia defixaria* (Walker, 1861)	woody	specialist	0	0	1	0
** Geometridae **	*Chiasmia hebesata* (Walker, 1861)	herb/woody	generalist	1	0	1	1
** Geometridae **	*Chlorissa amphitritaria* (Oberthür, 1879)	woody	generalist	1	0	0	1
** Geometridae **	*Chlorissa anadema* (Prout, 1930)	herb/woody	generalist	1	0	0	0
** Geometridae **	*Chlorissa obliterata* (Walker, 1863)	herb	generalist	1	0	0	0
** Geometridae **	*Chloroclystis v*-ata (Haworth, 1809)	herb/woody	generalist	1	1	0	0
** Geometridae **	*Cleora insolita* (Butler, 1878)	woody	generalist	1	0	0	0
** Geometridae **	*Comibaena delicatior* (Warren, 1897)	woody	generalist	1	0	0	1
** Geometridae **	*Costaconvexa caespitaria* (Christoph, 1881)	unknown	unknown	0	0	0	1
** Geometridae **	*Culpinia diffusa* (Walker, 1861)	herb/woody	generalist	1	0	0	0
** Geometridae **	*Cusiala stipitaria* (Oberthür, 1880)	woody	generalist	1	0	0	0
** Geometridae **	*Descoreba simplex* Butler, 1878	woody	generalist	1	0	0	0
** Geometridae **	*Dindica virescens* (Butler, 1878)	woody	specialist	1	1	0	0
** Geometridae **	*Ecliptopera capitata* (Herrich-Schäffer, 1840)	woody	specialist	1	0	0	0
** Geometridae **	*Ecliptopera umbrosaria* (Motschulsky, 1861)	woody	specialist	1	0	0	0
** Geometridae **	*Ecpetelia albifrontaria* (Leech, 1891)	woody	specialist	1	0	0	0
** Geometridae **	*Ectropis excellens* (Butler, 1884)	herb/woody	generalist	1	0	0	0
** Geometridae **	*Ectropis obliqua* (Prout, 1915)	herb/woody	generalist	1	0	0	0
** Geometridae **	*Epirrhoe supergressa* (Butler, 1878)	herb	specialist	1	0	0	0
** Geometridae **	*Euphyia cineraria* (Butler, 1878)	unknown	unknown	1	0	0	0
** Geometridae **	*Eupithecia interpunctaria* Inoue, 1979	herb	specialist	1	0	0	0
** Geometridae **	*Eupithecia mandschurica* Staudinger, 1897	unknown	unknown	1	0	0	0
** Geometridae **	*Eupithecia neosatyrata* Inoue, 1979	woody	specialist	1	0	0	0
** Geometridae **	*Evecliptopera illitata* (Wileman, 1911)	woody	specialist	1	0	1	1
** Geometridae **	*Gandaritis fixseni* (Bremer, 1864)	woody	generalist	0	0	0	1
** Geometridae **	*Geometra dieckmanni* Graeser, 1889	woody	specialist	0	0	0	1
** Geometridae **	*Heterophleps fusca* (Butler, 1878)	unknown	unknown	0	1	0	0
** Geometridae **	*Heterothera postalbida* (Wileman, 1911)	woody	specialist	1	0	0	0
** Geometridae **	*Hypomecis punctinalis* (Scopoli, 1763)	woody	generalist	1	0	0	0
** Geometridae **	*Idaea auricruda* (Butler, 1879)	unknown	unknown	1	1	0	1
** Geometridae **	*Idaea denudaria* (Prout, 1913)	unknown	unknown	1	0	0	0
** Geometridae **	*Idaea invalida* (Butler, 1879)	unknown	unknown	1	0	0	0
** Geometridae **	*Idaea jakima* (Butler, 1878)	unknown	unknown	1	0	0	0
** Geometridae **	*Idaea muricata* (Hufnagel, 1767)	unknown	unknown	1	1	0	1
** Geometridae **	*Idaea nielseni* (Hedemann, 1879)	unknown	unknown	0	0	1	0
** Geometridae **	*Idaea trisetata* (Prout, 1922)	unknown	unknown	1	0	0	0
** Geometridae **	*Jankowskia pseudathleta* Sato, 1980	woody	generalist	1	0	0	0
** Geometridae **	*Lamprocabera candidaria* (Leech, 1897)	woody	specialist	0	1	0	1
** Geometridae **	*Lassaba nikkonis* (Butler, 1881)	woody	generalist	1	0	0	0
** Geometridae **	*Lomographa bimaculata* (Fabricius, 1775)	woody	specialist	0	0	1	0
** Geometridae **	*Lomographa temerata* (Denis & Schiffermüller, 1775)	woody	specialist	1	1	1	1
** Geometridae **	*Macaria liturata* (Clerck, 1759)	woody	specialist	0	0	0	1
** Geometridae **	*Macaria shanghaisaria* Walker, 1861	woody	specialist	1	0	0	0
** Geometridae **	*Menophra senilis* (Butler, 1878)	herb/woody	generalist	1	1	0	0
** Geometridae **	*Metabraxas clerica* Butler, 1881	unknown	unknown	1	1	0	0
** Geometridae **	*Microcalicha sordida* (Butler, 1878)	woody	specialist	1	0	0	0
** Geometridae **	*Ninodes splendens* (Butler, 1878)	woody	specialist	1	0	0	0
** Geometridae **	*Orthonama obstipata* (Fabricius, 1794)	herb/woody	generalist	1	0	0	0
** Geometridae **	*Otoplecta frigida* (Butler, 1878)	woody	specialist	1	0	0	0
** Geometridae **	*Ourapteryx maculicaudaria* (Motschulsky, 1866)	woody	generalist	1	0	0	0
** Geometridae **	*Oxymacaria normata* (Alphéraky, 1892)	woody	specialist	1	0	0	0
** Geometridae **	*Pachista superans* (Butler, 1878)	woody	specialist	0	1	1	1
** Geometridae **	*Pasiphila excisa* (Butler, 1878)	woody	generalist	0	1	0	1
** Geometridae **	*Peratophyga hyalinata* (Kollar, 1844)	herb	specialist	1	0	0	0
** Geometridae **	*Petelia rivulosa* (Butler, 1881)	woody	specialist	1	0	0	0
** Geometridae **	*Petrophora chlorosata* (Scopoli, 1763)	herb	specialist	1	1	1	1
** Geometridae **	*Phthonosema tendinosaria* (Bremer, 1864)	herb/woody	generalist	1	1	1	1
** Geometridae **	*Pingasa alba* Swinhoe, 1891	woody	specialist	0	1	0	0
** Geometridae **	*Pingasa pseudoterpnaria* (Guenée, 1857)	woody	specialist	1	0	0	0
** Geometridae **	*Protoboarmia faustinata* (Warren, 1897)	woody	generalist	1	0	0	0
** Geometridae **	*Protoboarmia simpliciaria* (Leech, 1897)	woody	generalist	0	1	0	0
** Geometridae **	*Pseuderannis lomozemia* (Prout, 1930)	woody	generalist	1	0	0	0
** Geometridae **	*Pylargosceles steganioides* (Butler, 1878)	herb/woody	generalist	1	0	0	0
** Geometridae **	*Racotis petrosa* (Butler, 1879)	woody	specialist	1	0	0	0
** Geometridae **	*Scopula floslactata* (Haworth, 1809)	herb	generalist	0	1	0	0
** Geometridae **	*Scopula ignobilis* (Warren, 1901)	herb/woody	generalist	1	0	0	0
** Geometridae **	*Scopula impersonata* (Walker, 1861)	herb	specialist	1	0	0	0
** Geometridae **	*Scopula nigropunctata* (Hufnagel, 1767)	herb/woody	generalist	1	0	0	0
** Geometridae **	*Scopula nupta* (Butler, 1878)	unknown	unknown	1	0	0	0
** Geometridae **	*Scopula pudicaria* (Motschulsky, 1861)	herb	specialist	0	0	0	1
** Geometridae **	*Scopula semignobilis* Inoue, 1942	herb	specialist	1	0	0	0
** Geometridae **	*Spilopera debilis* (Butler, 1878)	woody	specialist	1	1	1	1
** Geometridae **	*Trichopteryx ussurica* (Wehrli, 1927)	unknown	unknown	0	1	0	0
** Geometridae **	*Tyloptera bella* (Butler, 1878)	woody	specialist	1	1	0	0
** Geometridae **	*Xanthorhoe biriviata* (Borkhausen, 1794)	herb	specialist	1	1	0	0
** Geometridae **	*Xerodes albonotaria* (Bremer, 1864)	herb/woody	generalist	1	0	0	0
** Geometridae **	*Xerodes rufescentara* (Motschulsky, 1861)	woody	generalist	1	0	0	0
** Geometridae **	*Xerodes semilutata* (Lederer, 1853)	woody	specialist	1	1	1	1
** Lasiocampidae **	*Dendrolimus spectabilis* (Butler, 1877)	woody	specialist	1	0	0	0
** Lasiocampidae **	*Euthrix albomaculata* (Bremer, 1861)	herb	specialist	1	0	0	0
** Lasiocampidae **	*Euthrix potatoria* (Linnaeus, 1758)	herb/woody	generalist	1	1	1	0
** Lasiocampidae **	*Kunugia undans* (Walker, 1859)	woody	generalist	0	1	1	1
** Lasiocampidae **	*Malacosoma neustrium* (Linnaeus, 1758)	woody	generalist	1	0	0	0
** Limacodidae **	*Narosoideus flavidorsalis* (Staudinger, 1887)	woody	generalist	1	1	0	1
** Limacodidae **	*Parasa consocia* Walker, 1863	woody	generalist	1	0	0	0
** Limacodidae **	*Parasa hilarula* (Staudinger, 1887)	woody	generalist	1	1	0	1
** Limacodidae **	*Phlossa conjuncta* (Walker, 1855)	unknown	unknown	1	0	0	0
** Noctuidae **	*Abrostola ussuriensis* Dufay, 1958	unknown	unknown	1	0	0	0
** Noctuidae **	*Acanthoplusia agnata* (Staudinger, 1892)	herb	generalist	0	0	1	0
** Noctuidae **	*Acronicta hercules* (Felder & Rogenhofer, 1874)	woody	specialist	0	0	1	0
** Noctuidae **	*Acronicta rumicis* (Linnaeus, 1758)	herb/woody	generalist	1	1	0	0
** Noctuidae **	*Actinotia intermediata* (Bremer, 1861)	herb	specialist	1	0	0	0
** Noctuidae **	*Adrapsa simplex* (Butler, 1879)	dead leaves	generalist	1	0	0	0
** Noctuidae **	*Agrotis exclamationis* (Linnaeus, 1758)	herb	generalist	1	0	1	1
** Noctuidae **	*Agrotis ipsilon* (Hufnagel, 1766)	herb/woody	generalist	0	0	1	0
** Noctuidae **	*Agrotis segetum* (Denis & Schiffermüller, 1775)	herb	generalist	1	0	0	0
** Noctuidae **	*Amphipoea ussuriensis* (Petersen, 1914)	unknown	unknown	1	0	0	0
** Noctuidae **	*Amphipyra pyramidea* (Linnaeus, 1758)	woody	generalist	1	0	0	0
** Noctuidae **	*Anachrostis nigripunctalis* (Wileman, 1911)	woody	specialist	1	1	0	0
** Noctuidae **	*Anacronicta caliginea* (Butler, 1881)	herb	specialist	1	0	0	0
** Noctuidae **	*Anacronicta nitida* (Butler, 1878)	herb	specialist	1	0	0	0
** Noctuidae **	*Anapamea incerta* (Staudinger, 1892)	unknown	unknown	1	0	0	0
** Noctuidae **	*Anaplectoides virens* (Butler, 1878)	herb	specialist	1	0	0	0
** Noctuidae **	*Anarta (Calocestra) trifolii* (Hufnagel, 1766)	herb	generalist	0	0	0	1
** Noctuidae **	*Anorthoa angustipennis* (Matsumura, 1926)	woody	generalist	1	0	0	0
** Noctuidae **	*Apamea aquila* (Donzel, 1837)	unknown	unknown	1	0	0	0
** Noctuidae **	*Apamea hampsoni* Sugi, 1963	unknown	unknown	1	1	0	0
** Noctuidae **	*Apamea sordens* (Hufnagel, 1766)	herb	specialist	0	0	0	1
** Noctuidae **	*Archanara resoluta* Hampson, 1910	herb	specialist	1	0	0	0
** Noctuidae **	*Athetis albisignata* (Oberthür, 1879)	herb	generalist	1	0	0	0
** Noctuidae **	*Athetis cinerascens* (Motschulsky, 1861)	unknown	unknown	1	0	0	0
** Noctuidae **	*Athetis dissimilis* (Hampson, 1909)	herb	generalist	1	0	0	0
** Noctuidae **	*Athetis lapidea* Wileman, 1911	unknown	unknown	1	1	1	1
** Noctuidae **	*Athetis lineosa* (Moore, 1881)	herb	generalist	1	0	0	0
** Noctuidae **	*Athetis stellata* (Moore, 1882)	herb	generalist	1	1	1	1
** Noctuidae **	*Aventiola pusilla* (Butler, 1879)	lichen/moss	generalist	1	0	0	0
** Noctuidae **	*Axylia putris* (Linnaeus, 1761)	herb	generalist	1	0	1	0
** Noctuidae **	*Bambusiphila vulgaris* (Butler, 1886)	herb	specialist	1	0	0	0
** Noctuidae **	*Blasticorhinus ussuriensis* (Bremer, 1861)	herb/woody	generalist	1	1	1	1
** Noctuidae **	*Bomolocha melanica* Sugi, 1959	unknown	unknown	1	0	0	0
** Noctuidae **	*Bomolocha stygiana* (Butler, 1878)	woody	specialist	1	0	0	1
** Noctuidae **	*Bomolocha zilla* (Butler, 1879)	unknown	unknown	1	0	0	0
** Noctuidae **	*Callopistria juventina* (Stoll, 1782)	herb	generalist	1	1	1	0
** Noctuidae **	*Callopistria placodoides* (Guenée, 1852)	herb	generalist	1	0	1	0
** Noctuidae **	*Callopistria repleta* Walker, 1858	herb	generalist	0	1	0	0
** Noctuidae **	*Calyptra thalictri* (Borkhausen, 1790)	herb	specialist	1	0	1	1
** Noctuidae **	*Catocala duplicata* Butler, 1885	woody	specialist	1	0	0	0
** Noctuidae **	*Catocala nagioides* Wileman, 1924	woody	specialist	1	0	0	0
** Noctuidae **	*Catocala nubila* Butler, 1881	woody	specialist	1	0	0	0
** Noctuidae **	*Chandata bella* (Butler, 1881)	herb	specialist	0	0	0	1
** Noctuidae **	*Chasminodes sugii* Kononenko, 1981	woody	specialist	1	0	0	0
** Noctuidae **	*Chorsia japonica* (Warren, 1912)	woody	specialist	0	0	0	1
** Noctuidae **	*Chrysodeixis acuta* (Walker, 1857)	herb/woody	generalist	1	0	0	0
** Noctuidae **	*Chrysorithrum amatum* (Bremer & Grey, 1853)	woody	specialist	1	0	0	1
** Noctuidae **	*Chytonix subalbonotata* Sugi, 1959	unknown	unknown	0	0	1	0
** Noctuidae **	*Cidariplura gladiata* Butler, 1879	unknown	unknown	1	0	0	0
** Noctuidae **	*Clavipalpula aurariae* (Oberthür, 1880)	woody	generalist	1	0	0	0
** Noctuidae **	*Conistra grisescens* Draudt, 1950	woody	generalist	1	0	0	0
** Noctuidae **	*Corgatha nitens* (Butler, 1879)	lichen/moss	generalist	0	1	0	0
** Noctuidae **	*Cosmia trapezina* (Linnaeus, 1758)	woody	generalist	1	0	0	0
** Noctuidae **	*Cryphia mitsuhashi* (Marumo, 1917)	lichen/moss	generalist	1	0	0	0
** Noctuidae **	*Cucullia perforata* Bremer, 1861	herb	specialist	0	1	0	0
** Noctuidae **	*Diarsia canescens* (Butler, 1878)	herb	generalist	1	1	1	1
** Noctuidae **	*Diarsia deparca* (Butler, 1879)	herb	generalist	0	0	0	1
** Noctuidae **	*Diarsia pacifica* Boursin, 1943	herb	generalist	1	0	0	0
** Noctuidae **	*Diarsia ruficauda* (Warren, 1909)	herb	generalist	1	0	0	0
** Noctuidae **	*Diomea cremata* (Butler, 1878)	lichen/moss	generalist	0	0	0	1
** Noctuidae **	*Dipterygina cupreotincta* Sugi, 1954	herb	specialist	1	0	0	0
** Noctuidae **	*Dypterygia andreji* Kardakoff, 1928	herb	specialist	1	0	0	0
** Noctuidae **	*Ercheia niveostrigata* Warren, 1913	herb	specialist	1	0	1	0
** Noctuidae **	*Ercheia umbrosa* Butler, 1881	woody	specialist	1	0	0	0
** Noctuidae **	*Ericeia pertendens* (Walker, 1858)	woody	specialist	0	1	0	0
** Noctuidae **	*Erygia apicalis* Guenée, 1852	herb/woody	specialist	0	0	1	0
** Noctuidae **	*Euplexia lucipara* (Linnaeus, 1758)	herb	generalist	0	1	1	0
** Noctuidae **	*Eutelia geyeri* (Felder & Rogenhofer, 1874)	woody	generalist	0	0	1	0
** Noctuidae **	*Euxoa karschi* (Graeser, 1889)	herb	specialist	1	0	0	0
** Noctuidae **	*Euxoa sibirica* (Boisduval, 1832)	herb	generalist	1	0	0	0
** Noctuidae **	*Gonitis mesogona* (Walker, 1858)	woody	generalist	1	0	0	0
** Noctuidae **	*Gortyna basalipunctata* Graeser, 1889	unknown	unknown	0	1	1	0
** Noctuidae **	*Gortyna fortis* (Butler, 1878)	herb	generalist	1	0	0	0
** Noctuidae **	*Gynaephila maculifera* Staudinger, 1892	lichen/moss	generalist	1	0	0	0
** Noctuidae **	*Harita belinda* (Butler, 1879)	woody	specialist	1	0	0	0
** Noctuidae **	*Heliothis maritima* Graslin, 1855	herb	generalist	0	0	1	0
** Noctuidae **	*Herminia arenosa* Butler, 1878	dead leaves	generalist	1	0	0	0
** Noctuidae **	*Herminia grisealis* (Denis & Schiffermüller, 1775)	dead leaves	generalist	1	0	0	0
** Noctuidae **	*Herminia tarsicrinalis* (Knoch, 1782)	dead leaves	generalist	1	0	0	0
** Noctuidae **	*Holocryptis ussuriensis* (Rebel, 1901)	unknown	unknown	1	0	0	0
** Noctuidae **	*Honeyania ragusana* (Freyer, 1844)	unknown	unknown	1	0	0	0
** Noctuidae **	*Hydraecia petasitis* Doubleday, 1847	herb	generalist	1	0	0	0
** Noctuidae **	*Hydrillodes morosa* (Butler, 1879)	dead leaves	generalist	1	1	1	1
** Noctuidae **	*Hypena amica* (Butler, 1878)	herb	specialist	0	1	0	0
** Noctuidae **	*Hypena kengkalis* Bremer, 1864	woody	specialist	1	0	0	0
** Noctuidae **	*Hypena* sp.	unknown	unknown	0	0	1	1
** Noctuidae **	*Hypena tristalis* Lederer, 1853	herb/woody	generalist	1	1	1	1
** Noctuidae **	*Hypena whitelyi* (Butler, 1879)	unknown	unknown	1	0	0	0
** Noctuidae **	*Hyperstrotia flavipuncta* (Leech, 1889)	dead leaves	generalist	1	1	1	1
** Noctuidae **	*Iambia japonica* Sugi, 1958	unknown	unknown	1	1	0	0
** Noctuidae **	*Idia curvipalpis* (Butler, 1879)	dead leaves	generalist	1	0	0	0
** Noctuidae **	*Koyaga falsa* (Butler, 1885)	herb	specialist	0	0	1	1
** Noctuidae **	*Koyaga numisma* (Staudinger, 1888)	unknown	unknown	1	0	0	0
** Noctuidae **	*Leucapamea kawadai* (Sugi, 1955)	unknown	unknown	1	0	0	0
** Noctuidae **	*Lygephila maxima* (Bremer, 1861)	herb	generalist	1	0	0	0
** Noctuidae **	*Mamestra brassicae* (Linnaeus, 1758)	herb	generalist	1	0	0	0
** Noctuidae **	*Melapia electaria* (Bremer, 1864)	herb	specialist	1	0	0	0
** Noctuidae **	*Metopta rectifasciata* (Ménétriès, 1863)	herb	specialist	1	1	0	1
** Noctuidae **	*Micardia argentata* Butler, 1878	herb	specialist	0	1	0	0
** Noctuidae **	*Micreremites pyraloides* Sugi, 1982	unknown	unknown	1	0	0	0
** Noctuidae **	*Microxyla confusa* (Wileman, 1911)	herb	specialist	1	0	0	0
** Noctuidae **	*Mocis ancilla* (Warren, 1913)	herb	specialist	1	0	0	0
** Noctuidae **	*Mocis annetta* (Butler, 1878)	herb/woody	specialist	1	0	1	0
** Noctuidae **	*Mosopia sordidum* (Butler, 1879)	unknown	unknown	1	0	0	0
** Noctuidae **	*Mythimna chosenicola* (Bryk, 1949)	unknown	unknown	1	0	0	0
** Noctuidae **	*Mythimna flavostigma* (Bremer, 1861)	unknown	unknown	1	0	1	1
** Noctuidae **	*Mythimna inanis* (Oberthür, 1880)	unknown	unknown	1	1	0	1
** Noctuidae **	*Mythimna matsumuriana* (Bryk, 1949)	herb	specialist	1	0	0	0
** Noctuidae **	*Mythimna obsoleta* (Hübner, 1803)	unknown	unknown	1	0	0	0
** Noctuidae **	*Mythimna pallens* (Linnaeus, 1758)	herb	generalist	1	0	0	0
** Noctuidae **	*Mythimna postica* (Hampson, 1905)	herb	specialist	1	0	0	0
** Noctuidae **	*Mythimna pudorina* (Denis & Schiffermüller, 1775)	herb	generalist	0	1	0	0
** Noctuidae **	*Mythimna radiata* (Bremer, 1861)	unknown	unknown	1	0	0	0
** Noctuidae **	*Mythimna rufipennis* Butler, 1878	unknown	unknown	1	0	1	1
** Noctuidae **	*Mythimna separata* (Walker, 1865)	herb	generalist	1	0	0	0
** Noctuidae **	*Mythimna turca* (Linnaeus, 1761)	herb	generalist	1	1	1	1
** Noctuidae **	*Naranga aenescens* Moore, 1881	herb	specialist	1	0	0	0
** Noctuidae **	*Niphonyx segregata* (Butler, 1878)	herb	specialist	1	0	0	0
** Noctuidae **	*Nycteola asiatica* (Krulikowski, 1904)	woody	generalist	1	0	0	0
** Noctuidae **	*Ochropleura plecta* (Linnaeus, 1761)	herb	generalist	1	0	0	0
** Noctuidae **	*Orthogonia sera* Felder & Felder, 1862	herb	specialist	1	0	0	0
** Noctuidae **	*Orthosia ella* (Butler, 1878)	herb	generalist	1	0	0	0
** Noctuidae **	*Orthosia gothica* (Linnaeus, 1758)	woody	generalist	0	1	0	0
** Noctuidae **	*Orthosia lizetta* Butler, 1878	woody	specialist	1	0	0	0
** Noctuidae **	*Oruza mira* (Butler, 1879)	dead leaves	generalist	1	0	0	0
** Noctuidae **	*Pangrapta lunulata* (Sterz, 1915)	woody	specialist	0	1	0	0
** Noctuidae **	*Pangrapta obscurata* (Butler, 1879)	woody	generalist	1	1	0	0
** Noctuidae **	*Pangrapta umbrosa* (Leech, 1900)	woody	specialist	1	0	1	0
** Noctuidae **	*Panolis japonica* Draudt, 1935	woody	specialist	1	0	0	0
** Noctuidae **	*Paracolax trilinealis* (Bremer, 1864)	unknown	unknown	1	0	0	0
** Noctuidae **	*Paracolax tristalis* (Fabricius, 1794)	dead leaves	generalist	0	1	0	1
** Noctuidae **	*Paragabara flavomacula* (Oberthür, 1880)	unknown	unknown	1	0	0	0
** Noctuidae **	*Perigrapha hoenei* Püngeler, 1914	woody	specialist	1	0	0	0
** Noctuidae **	*Phyllophila obliterata* (Rambur, 1833)	herb	specialist	1	1	1	1
** Noctuidae **	*Plusiodonta casta* (Butler, 1878)	woody	specialist	1	1	1	0
** Noctuidae **	*Prospalta cyclica* (Hampson, 1908)	unknown	unknown	1	0	0	0
** Noctuidae **	*Protodeltote distinguenda* (Staudinger, 1888)	herb	specialist	1	1	0	0
** Noctuidae **	*Protodeltote pygarga* (Hufnagel, 1766)	unknown	unknown	1	0	0	0
** Noctuidae **	*Pyrrhia umbra* (Hufnagel, 1766)	herb	generalist	1	0	0	0
** Noctuidae **	*Rhizedra lutosa* (Hübner, 1803)	herb	specialist	1	0	0	0
** Noctuidae **	*Rivula sericealis* (Scopoli, 1763)	herb	specialist	0	0	0	1
** Noctuidae **	*Rusicada privata* (Walker, 1865)	woody	specialist	0	0	1	0
** Noctuidae **	*Sarcopolia illoba* (Butler, 1878)	herb/woody	generalist	1	0	0	0
** Noctuidae **	*Schrankia separatalis* (Herz, 1905)	unknown	unknown	1	1	0	0
** Noctuidae **	*Sesamia confusa* (Sugi, 1982)	herb	specialist	1	0	0	1
** Noctuidae **	*Sesamia turpis* (Butler, 1879)	herb	specialist	1	0	0	0
** Noctuidae **	*Sidemia bremeri* (Erschoff, 1867)	unknown	unknown	0	1	1	0
** Noctuidae **	*Sineugraphe bipartita* (Graeser, 1889)	unknown	unknown	1	0	0	0
** Noctuidae **	*Sineugraphe bipartita* (Graeser, 1889)	unknown	unknown	0	1	0	0
** Noctuidae **	*Sineugraphe exusta* (Butler, 1878)	unknown	unknown	1	1	1	1
** Noctuidae **	*Sineugraphe oceanica* (Kardakoff, 1928)	herb	specialist	1	1	1	1
** Noctuidae **	*Sophta subrosea* (Butler, 1881)	unknown	unknown	1	0	0	0
** Noctuidae **	*Sphragifera sigillata* (Ménétriès, 1859)	woody	generalist	1	0	0	0
** Noctuidae **	*Spodoptera depravata* (Butler, 1879)	herb	specialist	1	0	0	0
** Noctuidae **	*Stenhypena nigripuncta* (Wileman, 1911)	lichen/moss	generalist	1	0	0	0
** Noctuidae **	*Stenoloba clara* (Leech, 1889)	unknown	unknown	0	0	1	0
** Noctuidae **	*Stenoloba jankowskii* (Oberthür, 1885)	unknown	unknown	0	0	0	1
** Noctuidae **	*Sugia erastroides* (Draudt, 1950)	unknown	unknown	1	0	0	0
** Noctuidae **	*Sugia idiostygia* (Sugi, 1958)	unknown	unknown	0	1	0	0
** Noctuidae **	*Sugia stygia* (Butler, 1878)	unknown	unknown	0	1	0	0
** Noctuidae **	*Sypnoides picta* (Butler, 1877)	woody	generalist	0	1	0	0
** Noctuidae **	*Telorta edentata* (Leech, 1889)	woody	generalist	0	0	1	0
** Noctuidae **	*Thyas juno* (Dalman, 1823)	woody	generalist	1	0	0	0
** Noctuidae **	*Trachea punkikonis* Matsumura, 1927	herb	specialist	1	0	0	0
** Noctuidae **	*Trachea tokiensis* (Butler, 1884)	herb	specialist	1	0	0	0
** Noctuidae **	*Treitschkendia tarsipennalis* (Treitschke, 1835)	dead leaves	generalist	1	0	0	0
** Noctuidae **	*Virgo datanidia* (Butler, 1885)	unknown	unknown	0	1	1	0
** Noctuidae **	*Xestia c-nigrum* (Linnaeus, 1758)	herb	generalist	1	0	0	0
** Noctuidae **	*Xestia ditrapezium* (Denis & Schiffermüller, 1775)	herb	generalist	1	0	0	0
** Noctuidae **	*Xestia efforescens* (Butler, 1879)	unknown	unknown	1	1	1	1
** Noctuidae **	*Xestia stupenda* (Butler, 1878)	herb	generalist	1	1	1	1
** Noctuidae **	*Zanclognatha lunalis* (Scopoli, 1763)	dead leaves	generalist	1	0	0	0
** Noctuidae **	*Zekelita plusioides* (Butler, 1879)	lichen/moss	generalist	1	0	0	0
** Nolidae **	*Earias pudicana* Staudinger, 1887	woody	generalist	1	1	0	0
** Nolidae **	*Gelastocera exusta* Butler, 1877	woody	generalist	1	0	0	0
** Nolidae **	*Gelastocera kotschubeji* Obraztsov, 1943	woody	generalist	1	0	0	0
** Nolidae **	*Nola taeniata* Snellen, 1874	unknown	unknown	0	1	0	0
** Nolidae **	*Nola trilinea* Marumo, 1923	herb	specialist	1	0	0	0
** Nolidae **	*Pseudoips prasinanus* (Linnaeus, 1758)	woody	specialist	1	0	1	0
** Notodontidae **	*Clostera anachoreta* Denis & Schiffermüller, 1775	woody	specialist	1	0	0	0
** Notodontidae **	*Cutuza straminea* (Walker, 1865)	herb	specialist	1	0	0	0
** Notodontidae **	*Epodonta lineata* (Oberthür, 1880)	woody	specialist	1	1	0	0
** Notodontidae **	*Fentonia ocypete* (Bremer, 1861)	woody	specialist	1	0	0	1
** Notodontidae **	*Furcula furcula* (Clerck, 1759)	woody	specialist	1	0	0	0
** Notodontidae **	*Gonoclostera timoniorum* (Bremer, 1861)	woody	specialist	1	0	0	0
** Notodontidae **	*Hexafrenum leucodera* (Staudinger, 1892)	woody	generalist	1	0	0	0
** Notodontidae **	*Hupodonta corticalis* Butler, 1877	woody	specialist	1	0	0	0
** Notodontidae **	*Mimopydna pallida* (Butler, 1877)	herb	specialist	1	0	0	0
** Notodontidae **	*Nerice bipartita* Butler, 1885	woody	specialist	0	1	0	0
** Notodontidae **	*Peridea gigantea* Butler, 1877	woody	specialist	1	0	0	0
** Notodontidae **	*Phalera flavescens* (Bremer & Grey, 1853)	woody	specialist	1	0	0	0
** Notodontidae **	*Phalerodonta manleyi* (Leech, 1889)	woody	specialist	0	1	0	0
** Notodontidae **	*Pheosiopsis cinerea* (Butler, 1879)	woody	specialist	1	0	0	0
** Notodontidae **	*Pterostoma gigantinum* Staudinger, 1892	woody	specialist	1	0	0	0
** Notodontidae **	*Shaka atrovittatus* (Bremer, 1861)	woody	specialist	0	1	1	0
** Notodontidae **	*Stauropus basalis* (Moore, 1877)	woody	generalist	1	0	0	1
** Notodontidae **	*Stauropus fagi* (Linnaeus, 1758)	woody	generalist	1	0	0	0
** Notodontidae **	*Syntypistis cyanea* (Leech, 1889)	woody	specialist	1	0	0	0
** Oecophoridae **	*Acryptolechia malacobyrsa* (Meyrick, 1921)	dead leaves	generalist	0	0	0	1
** Oecophoridae **	*Acryptolechia* sp. 1	unknown	unknown	0	1	1	0
** Oecophoridae **	*Periacma delegata* Meyrick, 1914	unknown	unknown	0	1	1	0
** Oecophoridae **	*Tyrolimnas anthraconesa* Meyrick, 1934	dead leaves	generalist	0	1	0	0
** Pyralidae **	*Acrobasis bellulella* (Ragonot, 1893)	woody	generalist	1	0	0	0
** Pyralidae **	*Acrobasis birgitella* (Roesler, 1975)	woody	specialist	1	0	0	0
** Pyralidae **	*Acrobasis ferruginella* Wileman, 1911	woody	specialist	1	0	0	0
** Pyralidae **	*Acrobasis squalidella* Christoph, 1881	woody	specialist	0	1	0	1
** Pyralidae **	*Addyme confusalis* Yamanaka, 2006	herb/woody	generalist	1	1	0	1
** Pyralidae **	*Assara funerella* (Ragonot, 1901)	woody	specialist	1	0	0	0
** Pyralidae **	*Ceroprepes ophthalmicella* (Christoph, 1881)	woody	specialist	1	0	0	0
** Pyralidae **	*Ceroprepes patriciella* Zeller, 1867	unknown	unknown	1	0	0	0
** Pyralidae **	*Ectomyelois pyrivorella* (Matsumura, 1899)	woody	specialist	1	0	0	0
** Pyralidae **	*Emmalocera venosella* (Wileman, 1911)	unknown	unknown	1	1	0	1
** Pyralidae **	*Endotricha consocia* (Butler, 1879)	unknown	unknown	0	1	0	1
** Pyralidae **	*Endotricha kuznetzovi* Whalley, 1963	dead leaves	generalist	1	1	1	0
** Pyralidae **	*Endotricha olivacealis* (Bremer, 1864)	dead leaves	generalist	1	1	0	1
** Pyralidae **	*Etielloides bipartitellus* (Leech, 1889)	unknown	unknown	1	0	0	0
** Pyralidae **	*Euzophera batangensis* Caradja, 1939	woody	generalist	1	0	0	0
** Pyralidae **	*Furcata dichromella* (Ragonot, 1893)	woody	specialist	1	0	0	0
** Pyralidae **	*Furcata hollandella* (Ragonot, 1893)	woody	specialist	1	0	0	0
** Pyralidae **	*Furcata pseudodichromella* (Yamanaka, 1980)	woody	specialist	1	0	0	0
** Pyralidae **	*Hypsopygia regina* (Butler, 1879)	woody	generalist	0	1	0	1
** Pyralidae **	*Lepidogma melanobasis* Hampson, 1906	unknown	unknown	0	1	0	1
** Pyralidae **	*Locastra muscosalis* (Walker, 1866)	woody	generalist	1	0	0	0
** Pyralidae **	*Maliarpha borealis* Sasaki, 2012	unknown	unknown	0	1	0	0
** Pyralidae **	*Nyctegretis trigangulella* (Hampson, 1901)	unknown	unknown	1	0	0	0
** Pyralidae **	*Oncocera bitinctella* (Wileman, 1911)	unknown	unknown	1	0	0	0
** Pyralidae **	*Oncocera semirubella* (Scopoli, 1763)	herb	specialist	1	1	1	1
** Pyralidae **	*Orthaga achatina* (Butler, 1878)	woody	generalist	0	0	0	1
** Pyralidae **	*Orthaga euadrusalis* Walker, 1859	woody	specialist	0	0	0	1
** Pyralidae **	*Ortholepis infausta* (Ragonot, 1893)	unknown	unknown	1	0	0	0
** Pyralidae **	*Orthopygia placens* (Butler, 1879)	unknown	unknown	1	0	0	0
** Pyralidae **	*Paraemmalocera gensanalis* (South, 1901)	unknown	unknown	1	0	0	0
** Pyralidae **	*Patagoniodes nipponellus* (Ragonot, 1901)	unknown	unknown	1	0	0	0
** Pyralidae **	*Phycitodes matsumurellus* (Shibuya, 1927)	unknown	unknown	0	1	0	0
** Pyralidae **	*Pseudacrobasis nankingella* Roesler, 1975	unknown	unknown	0	1	0	0
** Pyralidae **	*Quasipuer colon* (Christoph, 1881)	unknown	unknown	1	0	0	1
** Pyralidae **	*Sacada fasciata* (Butler, 1878)	woody	generalist	1	0	0	1
** Pyralidae **	*Salma amica* (Butler, 1879)	unknown	unknown	1	0	0	0
** Pyralidae **	*Salma elegans* (Butler, 1881)	herb/woody	generalist	1	1	0	0
** Pyralidae **	*Scirpophaga virginia* Schultze, 1908	unknown	unknown	1	0	0	0
** Pyralidae **	*Scirpophaga xanthopygata* Schawerda, 1922	unknown	unknown	1	0	0	0
** Pyralidae **	*Selagia spadicella* (Hübner, 1796)	unknown	unknown	0	0	0	1
** Pyralidae **	*Tegulifera bicoloralis* (Leech, 1889)	dead leaves	generalist	1	0	0	0
** Pyralidae **	*Termioptycha margarita* (Butler, 1879)	unknown	unknown	1	0	0	1
** Pyralidae **	*Trebania flavifrontalis* (Leech, 1889)	unknown	unknown	0	1	0	0
** Saturniidae **	*Actias aliena* (Butler, 1879)	woody	generalist	1	0	0	0
** Saturniidae **	*Saturnia japonica* (Moore, 1872)	woody	generalist	1	0	0	0
** Saturniidae **	*Saturnia jonasii* (Butler, 1877)	woody	generalist	0	1	1	1
** Sphingidae **	*Acosmeryx naga* (Moore, 1858)	woody	specialist	1	1	0	1
** Sphingidae **	*Agrius convolvuli* (Linnaeus, 1758)	herb	generalist	0	0	1	0
** Sphingidae **	*Ambulyx ochracea* Butler, 1885	woody	specialist	1	0	0	0
** Sphingidae **	*Ampelophaga rubiginosa* Bremer & Grey, 1853	woody	specialist	1	1	0	1
** Sphingidae **	*Callambulyx tatarinovii* (Bremer & Grey, 1852)	woody	specialist	1	0	0	0
** Sphingidae **	*Clanis bilineata* (Walker, 1866)	herb/woody	generalist	1	1	0	0
** Sphingidae **	*Deilephila askoldensis* (Oberthür, 1879)	herb	specialist	1	1	1	0
** Sphingidae **	*Deilephila elpenor* (Linnaeus, 1758)	herb	generalist	1	0	0	0
** Sphingidae **	*Marumba gaschkewitschii* (Bremer & Grey, 1853)	herb/woody	specialist	1	0	1	1
** Sphingidae **	*Marumba sperchius* (Ménétriès, 1857)	woody	specialist	1	0	0	0
** Sphingidae **	*Smerinthus planus* Walker, 1856	woody	generalist	1	0	0	0
** Sphingidae **	*Theretra japonica* (Boisduval, 1869)	herb/woody	generalist	1	0	1	1
** Thyrididae **	*Striglina cancellata* (Christoph, 1881)	woody	specialist	1	1	0	0
** Tortricidae **	*Aethes cnicana* (Westwood, 1854)	unknown	unknown	1	0	0	0
** Tortricidae **	*Aethes rectilineana* (Caradja, 1939)	unknown	unknown	1	0	0	0
** Tortricidae **	*Ancylis mandarinana* Walsingham, 1900	woody	specialist	1	0	0	0
** Tortricidae **	*Apotomis basipunctana* (Walsingham, 1900)	woody	specialist	0	0	0	1
** Tortricidae **	*Apotomis capreana* (Hübner, 1817)	woody	generalist	1	0	0	0
** Tortricidae **	*Apotomis geminata* (Walsingham, 1900)	woody	generalist	1	0	0	0
** Tortricidae **	*Archips audax* Razowski, 1977	herb/woody	generalist	1	0	0	0
** Tortricidae **	*Archips fuscocupreana* Walsingham, 1900	herb/woody	generalist	1	0	0	0
** Tortricidae **	*Archips ingentana* (Christoph, 1881)	herb/woody	generalist	1	0	0	0
** Tortricidae **	*Archips oporana* (Linnaeus, 1758)	woody	generalist	1	0	0	0
** Tortricidae **	*Archips semistructa* (Meyrick, 1937)	herb/woody	generalist	1	0	0	0
** Tortricidae **	*Choristoneura longicellana* (Walsingham, 1900)	woody	generalist	1	0	0	0
** Tortricidae **	*Cochylidia subroseana* (Haworth, 1811)	herb	specialist	1	0	0	0
** Tortricidae **	*Cydia danilevskyi* (Kuznetzov, 1973)	woody	specialist	1	0	0	0
** Tortricidae **	*Diplocalyptis congruentana* (Kennel, 1901)	herb	specialist	0	0	0	1
** Tortricidae **	*Epiblema autolitha* (Meyrick, 1931)	unknown	unknown	1	0	0	0
** Tortricidae **	*Epiblema foenella* (Linnaeus, 1758)	herb	specialist	1	1	1	1
** Tortricidae **	*Epinotia bicolor* (Walsingham, 1900)	woody	specialist	1	0	0	0
** Tortricidae **	*Epinotia majorana* (Caradja, 1916)	herb	specialist	1	0	0	0
** Tortricidae **	*Eucosma catharaspis* (Meyrick, 1922)	unknown	unknown	1	0	0	0
** Tortricidae **	*Eucosma denigratana* (Kennel, 1901)	unknown	unknown	1	0	0	0
** Tortricidae **	*Eucosma metzneriana* (Treitschke, 1830)	herb	specialist	1	0	0	0
** Tortricidae **	*Eucosma striatiradix* Kuznetzov, 1964	unknown	unknown	1	0	0	0
** Tortricidae **	*Eucosma tundrana* (Kennel, 1900)	unknown	unknown	0	1	0	0
** Tortricidae **	*Eudemis porphyrana* (Hübner, 1799)	woody	specialist	0	1	0	0
** Tortricidae **	*Eugnosta dives* (Butler, 1878)	herb	specialist	0	0	1	0
** Tortricidae **	*Eupoecilia ambiguella* (Hübner, 1976)	woody	generalist	0	0	0	1
** Tortricidae **	*Eupoecilia inouei* Kawabe, 1972	unknown	unknown	1	1	0	0
** Tortricidae **	*Gravitarmata margarotana* (Heinemann, 1863)	woody	specialist	1	0	0	0
** Tortricidae **	*Gynnidomorpha vectisana* (Humphreys & Westwood, 1845)	herb	specialist	1	0	0	0
** Tortricidae **	*Gypsonoma dealbana* (Frölich, 1828)	woody	generalist	0	0	0	1
** Tortricidae **	*Hedya auricristana* (Walsingham, 1900)	woody	generalist	1	0	0	0
** Tortricidae **	*Lobesia reliquana* (Hübner, 1776)	unknown	unknown	1	0	0	0
** Tortricidae **	*Matsumuraeses phaseoli* (Matsumura, 1900)	herb	specialist	1	0	0	0
** Tortricidae **	*Neoanathamna nipponica* (Kawabe, 1976)	dead leaves	generalist	0	0	0	1
** Tortricidae **	*Neocalyptis angustilineata* (Walsingham, 1900)	dead leaves	generalist	1	0	0	0
** Tortricidae **	*Olethreutes examinata* (Falkovitsh, 1966)	unknown	unknown	1	0	0	0
** Tortricidae **	*Pandemis dumetana* (Treitschke, 1835)	herb/woody	generalist	1	1	1	1
** Tortricidae **	*Phaecasiophora roseana* (Walsingham, 1900)	woody	specialist	1	1	0	1
** Tortricidae **	*Phtheochroa pistrinana* (Erschoff, 1877)	herb	specialist	1	0	0	1
** Tortricidae **	*Ptycholoma lecheana* (Linnaeus, 1758)	woody	generalist	1	0	0	0
** Tortricidae **	*Saliciphaga acharis* (Butler, 1879)	woody	specialist	1	0	0	0
